# A novel integration of online and flipped classroom instructional models in public health higher education

**DOI:** 10.1186/1472-6920-14-181

**Published:** 2014-08-29

**Authors:** Lindsay P Galway, Kitty K Corbett, Timothy K Takaro, Kate Tairyan, Erica Frank

**Affiliations:** Faculty of Health Sciences, Simon Fraser University, Burnaby, BC V5A 1S6 Canada; School of Public Health and Health Systems, University of Waterloo, Waterloo, Ontario N2L 3G1 Canada; School of Population and Public Health, University of British Columbia, Vancouver, BC V6T 1Z3 Canada

**Keywords:** Flipped classroom, Blended learning, E-learning, Public health education, Master of Public Health, Environmental and occupational health

## Abstract

**Background:**

In 2013, a cohort of public health students participated in a ‘flipped’ Environmental and Occupational Health course. Content for the course was delivered through NextGenU.org and active learning activities were carried out during in-class time. This paper reports on the design, implementation, and evaluation of this novel approach.

**Methods:**

Using mixed-methods, we examined learning experiences and perceptions of the flipped classroom model and assessed changes in students' self-perceived knowledge after participation in the course. We used pre- and post-course surveys to measure changes in self-perceived knowledge. The post-course survey also included items regarding learning experiences and perceptions of the flipped classroom model. We also compared standard course review and examination scores for the 2013 NextGenU/Flipped Classroom students to previous years when the course was taught with a lecture-based model. We conducted a focus group session to gain more in-depth understanding of student learning experiences and perceptions.

**Results:**

Students reported an increase in knowledge and survey and focus group data revealed positive learning experiences and perceptions of the flipped classroom model. Mean examination scores for the 2013 NextGenU/Flipped classroom students were 88.8% compared to 86.4% for traditional students (2011). On a scale of 1–5 (1 = lowest rank, 5 = highest rank), the mean overall rating for the 2013 NextGenU/Flipped classroom students was 4.7/5 compared to prior years’ overall ratings of 3.7 (2012), 4.3 (2011), 4.1 (2010), and 3.9 (2009). Two key themes emerged from the focus group data: 1) factors influencing positive learning experience (e.g., interactions with students and instructor); and 2) changes in attitudes towards environmental and occupation health (e.g., deepened interest in the field).

**Conclusion:**

Our results show that integration of the flipped classroom model with online NextGenU courses can be an effective innovation in public health higher education: students achieved similar examination scores, but NextGenU/Flipped classroom students rated their course experience more highly and reported positive learning experiences and an increase in self-perceived knowledge. These results are promising and suggest that this approach warrants further consideration and research.

## Background

The rapid increase in Internet access and advances in online technology over the last decade present an opportunity to rethink the way we teach and learn in the context of public health higher education. The flipped classroom instructional model (also known as the inverted classroom) has emerged as a promising alternative to conventional lecture-based teaching as it offers a framework for integrating emerging online learning technologies with active and collaborative learning. The flipped classroom model is a type of blended learning where in-class learning is integrated with online learning experiences [1, 2]. A meta-analysis from the United States Department of Education in 2010 showed that blended learning, such as the flipped classroom, is more effective than either face-to-face (i.e. lecture-based instruction) or online learning alone [3]. This meta-analysis was focused on K-12 education. There has been limited research exploring the flipped classroom model in the context of higher education (and none in the realm of public health higher education) highlighting an important knowledge gap. In an era of rising education costs and declining public funding for higher education, innovative approaches that foster positive learning experiences while taking advantage of emerging technologies and use both student and instructor time more efficiently are called for [4].

The defining characteristic of the flipped classroom instructional model is that content and material are delivered primarily outside of the classroom while in-class time is used "to work through problems, advance concepts, and engage in collaborative learning" [5]. Using online educational technologies to deliver content and material outside of the classroom frees up in-class time for active and collaborative application of content with the support of classmates and the instructor [2]. This model is designed to allow students to independently engage with materials on their own time and at their own pace, shifts focus from the instructor to the learner, and promotes active learning and problem-solving.

The flipped classroom model involves more than shifting content delivery outside of class time [6]. It represents a broader shift in how we think about the learning process. It is grounded in several interconnected theories of learning and pedagogy. The explicit attention to interactive and collaborative learning draws on Piaget’s theory of active learning which highlights that learning occurs when we act on and apply new ideas and concepts [7]. In terms of Bloom’s influential (revised) taxonomy of thinking and learning, the flipped classroom enables both higher and lower levels of cognitive work [8, 9]. More specifically, students do lower level cognitive work, i.e., the acquisition of knowledge, independently and outside of class while higher-order cognitive work including knowledge application, analysis, and synthesis occurs during class time with the support of peers and instructors. In our application of the flipped classroom design within the context of graduate level public health education, we also draw on Mesirow’s theory of transformative adult learning [10] and Habermas’ related theory of knowledge and human interests [11]. We therefore integrate reflection, a key aspect of learning according Mesirow and an essential type of knowledge according to Habermas, into the course to complement the online content delivery and the in-class application of knowledge.

There is no single or standard way to design and implement the flipped classroom instructional model in practice [6, 12]. The means of delivering content and the ways in which face-to-face class time is used will vary with the characteristics of the students, background of the instructor, available resources and the subject matter. To date, electronic video-recordings (vodcasts) and podcasts have been the primary means of content delivery, however online courses can also be used for this purpose. In our application of the flipped classroom model, we have used an environmental and occupational health course offered by NextGenU.org, a portal to free and accredited higher education [13]. Partnering with universities, professional societies, and government organizations, NextGenU creates online courses referred to as DOOHICHEs (Democratically-Open Outstanding Hybrids of Internet-aided, Computer-aided, and Human-aided Education, pronounced as "doohickey"). These DOOHICHEs are competency-based and include high-quality learning resources, online peer activities and discussion forums, and quizzes. As of February 22^nd^ 2014, NextGenU has over 2,000 students registered in 105 countries, and has 130 trainings in development, primarily focusing on health sciences and public health education. The ways in which NextGenU builds on and differs from traditional education and Massive Open Online Courses can be reviewed at http://www.NextGenU.org.

The goal of this study was to examine the impact of this NextGenU/Flipped classroom instructional model on self-perceived environmental and occupational health (EOH) knowledge and student learning experiences and perceptions of the instructional model. Our specific objectives were: 1) to design and implement a master’s level environmental and occupational health course (EOHC) integrating a NextGenU DOOHICHE and the flipped classroom model, 2) to assess changes in students' self-reported EOH knowledge after participating in the course, and 3) to understand student learning experiences and perceptions of this NextGenU/Flipped classroom model.

## Methods

### Course design and implementation

The first author designed the EOH DOOHICHE for NextGenU in 2012. The content and structure of the DOOHICHE was based on core Master of Public Health competencies developed by the Association of Schools of Public Health (APSH) [14, 15] and the Association of Schools of Public Health in the European Region (ASPHER) [16, 17]. These core competencies "delineate fundamental knowledge, attitudes, and skills that every MPH student, regardless of their major field, should possess upon graduation" [18]. The final version was reviewed by an advisory committee of environmental health professionals, and endorsed by the International Society of Doctors for the Environment, Physicians for Social Responsibility, and Simon Fraser University’s Faculty of Health Sciences.

NextGenU’s EOH DOOHICHE can be accessed for free in its entirety at http://www.NextGenU.org. Each of the course’s nine modules (see Table 1), offered students the opportunity to learn content and material by engaging with a diversity of learning material (e.g., reports, journal articles, videos, websites) from reputable sources including universities, governments, professional societies, and peer-reviewed publications. The online course platform also included discussion forums, peer-to-peer activities, quizzes, and additional learning resources for those students wanting to explore certain subject areas more deeply. In-class sessions were held every other week, which gave students two weeks to complete the assigned modules. Students were assigned either one or two modules for each two-week period. Within the two-week period, students could move through the materials at their own pace. After engaging with the learning materials and completing peer activities in a given module, and before coming to the in-class sessions, students completed a quiz that helped students and the instructor identify poorly-understood areas.

**Table 1:** **Overview of the course**

Target participants	MPH students
**Description of the course**	Students will gain familiarity with fundamental principles and general areas of knowledge that are important to the broad field of environmental health. Students will learn about approaches and tools used to recognize, assess, and manage environmental and occupational health hazards. This course also aims to expose students to numerous environmental and occupational health issues and to encourage critical thinking and reflection on these issues; we will consider what can be done about environmental and occupational health issues to ultimately protect and promote health and well-being. Finally, this course aims to inspire interest in the role of the environment in promoting and maintaining the health of populations in both local and global settings.
**Online learning modules from NextGenU DOOHICHE** ^**1**^ **:**	
Module 1:	Introduction to environmental and occupational health
Module 2:	Environmental and occupational hazards and their effects on human health and ecosystems
Module 3:	Principles of exposure assessment
Module 4:	Toxicology and epidemiology in environmental health
Module 5:	Risk assessment: Concepts and application
Module 6:	Risk management, communication, and regulation
Module 7:	Susceptibility, vulnerability, and inequality in environmental health
Module 8:	Environmental and occupational health case studies
Module 9:	Emerging perspectives in environmental health
**Learning activities used during class time:**	
Module 1:	Linking thinking for environmental health
Module 2:	Exposure assessment in action
Module 3:	Shipbreaking in Alang, India
Module 4 and 5:	Toxicology, epidemiology, and risk assessment problem set
Module 6:	Class debate- Should Canada apply the precautionary principle in environmental health policy and decision-making
Module 7:	A closer look at Air Quality Guidelines
Module 8 and 9:	Presentation and discussion of environmental health case studies
**Evaluation:**	
Final grades were based on:	1) A final exam consisting of multiple choice and short answer application questions
	2) Four graded reflective responses written throughout the semester
	3) In-class and online participation
	4) A final group project exploring a selected environmental or occupational health issue

^**1**^The online Learning modules in the NextGenU DOOHICHE can be accessed in full at http://www.nextgenu.org/course/view.php?id=52#0.

The general routine of in-class time included a mini-lesson addressing concepts or aspects that were identified as challenging by students, along with a brief question and answer period to give students the opportunity to clarify any remaining aspects from the assigned modules. The remaining in-class time (90 minutes) was used to carry out active learning activities. The learning activities varied for each in-class session. Examples include: a toxicology problem set that students worked on in pairs; an occupational health case-study examined in small groups; and a whole-class debate on the topic of environmental health decision-making (see Table 2 for a description of three in-class activities). Over the course of the 13-week semester there were eight in-class sessions that ran for two hours.

**Table 2:** **Description of a selection of in-class learning activities**

**Example 1**	
**Activity name:**	Linking thinking for environmental health
**Summary of activity:**	Each student selects a health issue of interest. Working on their own, students create an influence diagram on a large blank piece of paper that explicitly illustrates the links between their selected health issue and the environment or environmental factors. In pairs, the students describe their influence diagram to one another. Finally, the instructor facilitates a discussion around the following questions "Was it difficult to integrate the environment and/or environmental factors into the influence diagram for your selected health issue? Why or why not?"
	Adapted from [19]
**Example 2**	
**Activity name:**	Shipbreaking in Alang, India
**Summary of activity:**	Before coming to class, students are instructed to watch the documentary ‘Shipbreakers’. This documentary acted as background knowledge to complete the case-study described below. Students work on the case-study in groups of 3 or 4.
	*You and your co-workers from Workplace Health Without Borders have recently returned from a visit to the shipbreaking port in Alang, India (the movie that you watched before coming to class this week served as your visit). Although it was a short trip, while in Alang you had the opportunity to observe the work setting and living conditions as well as to speak with many shipbreakers, port owners, the doctor, and other members of the community. Upon your return, your boss has asked for a review of what you saw and learned while visiting Alang. Specifically, she would like you and your colleagues to create a presentation that addresses the following questions:*
	*1. Who works in the shipbreaking yards? Describe relevant characteristics of workers in the shipbreaking yards.*
	*2. What are the major health hazards associated with shipbreaking identified during your visit?*
	*3. Use the following classification scheme (from the Canadian Centre for Occupational Hazards and Safety) to classify the hazards you have identified as: a) Biological; b) Chemical; c) Ergonomic (i.e. repetitive strain injury); d) Physical (noise, radiation); e) Psychosocial.*
	*4. For the chemical hazards identified, note the potential health implications using information provided by the Agency for Toxic Substances and Disease Registry at the following website:* http://www.atsdr.cdc.gov/toxfaqs/index.asp.
	*5. Drawing on your experience in Alang, what are possible research activities, interventions, or policy changes that our organization could implement to improve the current situation in Alang? Be sure to provide ample justification for your proposed research, intervention, or policy ideas.*
**Example 3**	
**Activity name:**	Toxicology, epidemiology, and risk assessment problem set
**Summary of activity:**	Students work in pairs to solve problems applying toxicology, epidemiology, and risk assessment concepts.

An additional requirement was writing four reflective responses. Students were instructed to reflect on the meaning and importance of the course material from their own perspective, to make links between the various aspects of EOH within the course, and to make links to the student’s other public-health-related interests. The reflective responses acted as an ongoing conversation between the instructor and the students, and were also used by the instructor to identify areas of particular interest among the group and to influence and inform the direction of in-class activities.

Finally, students completed a final exam at the end of the semester that included multiple choice and short answer questions.

### Study participants

Study participants were MPH students (n = 11) enrolled in the 2013 spring session of an EOHC at a Canadian university. All participants were graduate students in their first or second year of study in the MPH program. An EOHC is offered in the second year of study and is a required core course of the MPH degree. Primary areas of interest among the students within the broad field of public health ranged from gender and health to social inequities and health. None of the students were focused specifically on environmental or occupational health nor enrolled in the EOH concentration of the MPH program. All of the students in the class agreed to participate in the study.

### Evaluation and analysis

We used a mixed-method approach including a pre- and post-survey, comparisons of overall course ratings and examination scores across years, and a post-course focus group session. A mixed-methods approach was used selected surveys are an effective tool for assessing pre and post student knowledge while focus groups are useful for gaining a more in-depth understanding of student perceptions and are also convenient and appropriate for non-sensitive topics.

**Figure 1:**
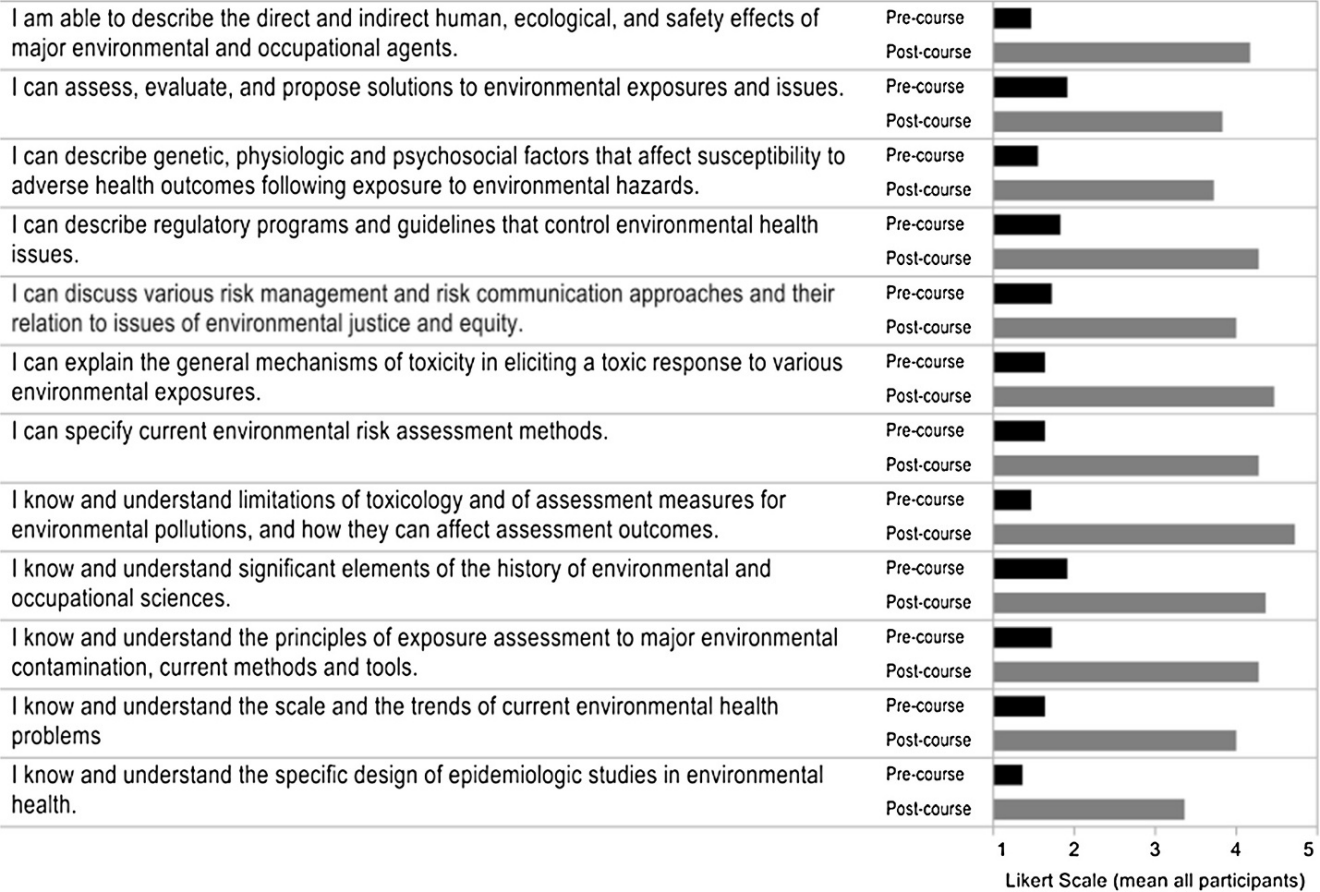
**Comparison of pre- and post-course self-perceived knowledge of EOH competencies.**

Pre- and post-course survey instruments were developed by the course instructor and reviewed by three colleagues. Participants electronically completed the pre-course survey one week prior to the beginning of the course and the post-course survey one week following course completion.To measure self-perceived knowledge, students were asked to rate their knowledge regarding EOH competencies. Students rated their knowledge relating to each competency (12 in total, see Figure 1) on a 5-point Likert scale (1 = strongly disagree; 5 = strongly agree). The self-perceived knowledge questions were identical in the pre- and post-course survey instruments. The post-course survey instrument also contained 10 items pertaining to student learning experiences and perceptions of the flipped classroom, and one open-ended question where students were asked for general comments and/or feedback.

To determine whether there was a statistically significant change in self-perceived EOH knowledge following participation in the class we used the Wilcoxon signed-rank test for paired data (5% significance level). This non-parametric test was selected because the data was non-normally distributed and ranked, and due to the small sample size. Descriptive statistics were used to report items pertaining to learning experiences and student perceptions of the flipped classroom model as measured in the post-course survey. For reporting of these items, agreement (strongly agree and agree) and disagreement (strongly disagree and disagree) were combined.

We also compared overall course ratings and examination scores for the NextGenU/Flipped classroom students against course ratings and examination scores for student who took a lecture-based EOHC in previous years. Specifically, we compared overall course ratings collected by the university from the 2013 students (n = 9 who participated in the course and completed the standard course review) with overall course ratings for students from 2012, 2011, 2010, and 2009 (n = 130). To compare examination scores, we looked at scores from the course exam for the 2013 NextGenU/Flipped classroom students (n = 11) compared to scores for the 2011 lecture-based EOHC students (n = 22). Examination result data were only available for 2011. The exam contained 11 short answer questions, and was marked by the same individual. A Mann–Whitney U test (5% significance level) was used to assess whether there was a statistically significant difference in the examination scores for the 2013 vs. the 2011 students.

Finally, to gain a more in-depth understanding of student perceptions of the NextGenU/Flipped classroom model, we (LPG) collected, audiotaped, and transcribed qualitative data from one 40-minute focus group session with all students in the course after the final in-class session. All focus group participants reviewed and signed a consent form. Open-ended queries addressed learning experiences and perceptions of the flipped classroom model in general. The data were analyzed using qualitative thematic analysis, selected because of the exploratory and descriptive nature of this study. First, the transcribed text was read in its entirety to get a general overview and sense of the data. During a second reading of the transcript, the data were coded to identify and explore themes. Major themes were therefore derived inductively from the data. Data were then summarized and representative excerpts from the focus group were identified to illustrate the major themes.

All data collection was completed by the end of the 2013 spring session of the university. Statistical analyses were conducted using R statistical software version 2.13.0. Given the manageable length of the single focus group session, we used Microsoft Word to analyze the qualitative data [20].

Ethics approval was obtained from The University of British Columbia Ethics Committee. Recruitment was conducted by the lead author (LPG) during the first class session. All participants gave written informed consent and were assured of confidentiality and anonymity. Also, the voluntary nature of the study was underscored, and it was made clear to students that they could end their participation in the study at any point and that their marks in the class would not be affected by their decision to participate in the study.

## Results

### Self-perceived knowledge assessment

The response rate for the pre-and post-course survey was 100%. A comparison of self-perceived knowledge of EOH competencies before and after the course is presented in Figure 1. A statistically significant increase was found in students’ self-perceived knowledge for every competency (p-value < 0.05).

### Students’ perceptions of the NextGenU/flipped classroom model

Our results show that student perceptions of the course and the NextGenU/Flipped classroom instructional model were highly favorable overall. In response to the item "In the future, would you rather take a ‘flipped’ course than a traditional lecture-based course", 82% of students agreed or strongly agreed. All students agreed or strongly agreed with the statement, "The flipped classroom model was a different learning experience than other MPH courses." Also, the use of a NextGenU DOOHICHE for the online delivery of content and materials was favorably received. In response to the item, "I was comfortable with self-directed online learning through NextGenU", all students either agreed or strongly agreed (see Table 3 for a summary of results from the post-course survey).

**Table 3:** **Descriptive statistics of post-course survey items focusing on learning experience and perceptions**

Survey item	Agree/strongly agree (%)	Disagree/strongly disagree (%)	Neutral (%)
I was comfortable with self-directed learning through NextGenU.	100	0	0
The online learning materials contributed to my learning.	100	0	0
I completed the activities and learning materials before in-class sessions.	100	0	0
The quizzes encouraged completion of the online learning materials.	82	0	18
In-class learning activities complemented online self-directed learning.	100	0	0
The reflective responses contributed to my learning.	91	0	9
Interaction with my instructor and other classmates contributed to my learning.	100	0	0
The flipped classroom model (online learning plus in-person classroom interaction and problem-solving) was a different learning experience than other MPH courses.	100	0	0
The flipped classroom model enabled more interaction with my instructor and classmates than did other MPH courses.	82	0	18
In the future, I would rather take a ‘flipped’ course (blended online learning plus in-persons classroom interaction and problem-solving) than a traditional (lecture-based) course.	82	9	9

*NB: Students were asked to react to statements on a 5-point Likert scale where 1 = "Strongly disagree" and 5 = "Strongly agree." For reporting of these survey items, agreement (strongly agree and agree) and disagreement (strongly disagree and disagree) were combined.*

In response to the only open-ended question in the post-course survey "Do you have any additional feedback/comments about the flipped classroom model?" students provided additional generally-positive feedback. One participant wrote: *"I really enjoyed the structure and content of this course. I would say it was one of the best courses I’ve taken during the MPH! Also, it changed my perspective on online learning. I never thought online learning would be beneficial for me, but I will definitely consider it if I decide to do continued studies later on."* A second student wrote, *"It was a great class and ignited an interest in enviro health that was not previously there*!"

### Comparison of overall course ratings and examination scores

The 2013 NextGenU/Flipped Classroom students rated their overall course experience more highly than those students who took the EOHC in previous years when a lecture-based model was used. On a scale of 1–5 (1 = lowest rank; 5 = highest rank), the mean overall rating for the course in 2013 was 4.7/5 vs. prior years’ overall ratings of 3.7 (2012), 4.3 (2011), 4.1 (2010), and 3.9 (2009). We compared examination scores for the 2013 NextGenU/Flipped classroom students to those for the 2011 lecture-based EOHC students. The mean test score was 88.8% for the 2013 students and 86.4% for the 2011 students. A Mann–Whitney U test revealed no statistically significant difference (p = 0.72) between examination scores across the two groups .

### Key themes from focus group data

Qualitative thematic analysis of the focus group data turned up two major themes: factors influencing positive learning experiences and changes in attitudes towards environmental and occupational health. These themes are discussed below along with excerpts from participants’ responses.

Students discussed multiple factors that contributed to their positive learning experiences*.* High amounts of interaction with other students and the instructor, small class size, the use of active in-class learning activities and reflective responses, and engagement with content online before attending in-class sessions were all positively-cited aspects of the course and its design. Students commented that the class size and the flipped model contributed to a more interactive learning environment and greater interaction with the instructor and their peers. This was recognized as deficient in previous courses: *"…the discussions that we were able to have in class, by having a small class, that what we were able to get out of an in-class session was a lot more than a 3 hour lecture once per week of fifty people."* Several students also noted that they were unsure whether the flipped classroom model would be as effective with a larger class. For example, one student stated, "*So if they are just going to try and flip i,t then just throw 50 people in there, I don’t know if it would work*."

The focus group also highlighted the contribution of reflective responses to positive learning experiences. They allowed students to make connections between various aspects of the course with other areas of public health and student lives in general. One student stated: "*And I found I would be like noticing environmental health news articles or stuff coming up, and you would think ‘Oh! I could really try and incorporate this into my reflection’*. A second student followed up on this comment stating: *"Ya – that is what I found. I found the reflections really encouraged me to be thinking about the course beyond the content, like beyond the actual readings because you don’t really reflect about readings a lot of the time. But I was drawing on other things, and it kind of encouraged me to make those connections, those bigger connections."*

Students also had positive comments about applying content through in-class learning activities. Students reported that the variety of learning activities used in the in-class sessions contributed to positive learning experiences. One student commented, *"So I feel like I took in a lot of it, so that when I came to class I was cementing the knowledge that I just read."* Another student said, *"every week there was something that was fresh and new and… interactive and I appreciated that."*

Finally, several students noted that they were much more likely to complete readings and engage with content before coming to class in the flipped classroom model than the traditional lecture-based model. One student stated, *"it is really easy to justify that you don’t have time to do readings, but for this set-up I think that is was just like, something I needed to get done."*

When students began this course, there was very little interest in the field of environmental health and occupational health in the group. For example, none of the students were part of the EOH concentration option available for the MPH students at the test-site university. The majority of students noted that they would not have taken the course had it not been a required core course for their degree. However, a major theme that emerged from the focus group was that this course contributed to greater interest in environmental health and environmental health issues in general. One student noted about the course: "*It gave me a huge appreciation for environmental health, it really did truly."* A second student agreed and stated: *"I feel like I got a tremendous appreciation for environmental health and I got a really good grounding in environmental and occupation health and so that is why, you know, I really really liked the course."*

## Discussion

This paper reported on the design, implementation, and evaluation of a master’s level EOHC that integrated two emerging instructional models: NextGenU’s DOOHICHE and the flipped classroom. Our results suggest that this innovative approach fostered learning and provided positive learning experiences for this small group of graduate students. To quote one of the students, *"This was a successful experiment!"* Students also expressed a preference for the flipped classroom instructional model over the traditional lecture-based model, with most (82%) agreeing that, "In the future, I would rather take a ‘flipped’ course (blended online learning plus in-persons classroom interaction and problem-solving) than a traditional (lecture-based) course. In addition, the instructor (LPG) had positive experiences regarding the design, implementation, and overall outcomes of the course.

This instructional model should be considered for more widespread experimentation in the context of public health higher education and beyond. It is worth noting that the flipped classroom, blended learning, and NextGenUs DOOHICHEs are three of many emerging approaches that are student-centered, promote application and collaboration, and optimize face-to-face time and complementary educational technologies [6]. We urge instructors interested in moving away from conventional lecture-based teaching to consider the wide range of instructional models available and to select the model which bests suits the course content, student needs, and available resources to optimally facilitate learning.

For those instructors considering applying the flipped classroom instructional model, we caution that ‘flipping’ the classroom is not simply about shifting lectures outside of the classroom. Content delivery is "just one small piece of the overall learning experience…" [21]. ‘Flipping’ the classroom involves seeing students as active learners, shifting control of both learning and the classroom from the instructor to the students; it should promote a focus on higher-order cognitive work. Additionally, research shows that, "for blended learning environments to be successful, it is important to structure the face-to-face and the online portions of the learning experience so that they coherently support one another" [22]. Educators should think purposefully about course design, develop effective learning activities that engage students, encourage reflections, and complement online content, and take maximal advantage of invaluable face-to-face class time.

Further, we encourage instructors to think creatively about how to use emerging educational technologies in their teaching. For this work, we have employed a NextGenU DOOHICHE to deliver content online, but other opportunities for innovation in blended learning also exist. The use of open online courses within the flipped classroom, and blended learning more broadly can reduce the burden of course re-design on instructors and institutions by most-fully utilizing already-existing and free courses.

Although we agree with others who suggest that that the flipped classroom model has the potential to influence the landscape of higher education [23–25], we also note that more research is needed to evaluate the impacts of this model on teaching and learning experiences and to better understand the specific characteristics of ‘flipped’ courses that lead to positive impacts. It is also important for instructors to share their experiences and course design to ensure that we are collectively capitalizing on lessons learned in implementation. Finally, more research is needed to understand the role of reflection in the flipped classroom instructional model. Reflection which plays a key role in adult learning, has not been explicitly considered within the flipped classroom model [10]. Students in the flipped classroom environments "need to have more space to reflect on their learning activities so that they can make necessary connections to course content" [1]. Qualitative evidence from this small cohort, as well as the opinions of the course instructor, suggest that reflection is an important yet overlooked element of applying the flipped classroom model.

There are limitations that should be noted. We cannot exclude the possibility that students answered questions in a socially desirable way nor that the positive perceptions of this instructional model were attributable to it being a different experience than other courses offered to the students. A major limitation of this study is the small sample size. As this was a pilot study, the sample size was simply the number of students that participated in the class. Finally, comparisons of overall course ratings and examination scores should be interpreted with caution. Although the courses were designed around the same MPH core competencies, there were important differences across years that we have not controlled for in the design or analysis. Specifically, different instructors taught the course, class sizes were higher in previous years (as high as 50) compared to 11 in the 2013, and reflection was only included as part of the curriculum in the flipped classroom EOHC. These factors could certainly influence both overall course ratings and examination scores.

## Conclusion

The lecture-based teaching model continues to dominate higher education despite massive advances in online access and technology and developments in pedagogical theory [26, 27]. Our results show that the flipped classroom model can have positive impacts on learning and learning experiences in public health higher education. Our data suggest that the use of a DOOHICHE from NextGenU was an effective and efficient means of content delivery and could facilitate more widespread application of the flipped classroom in public health higher education.

## Abbreviations

ASPH: Association of Schools of Public Health
ASPHER: Association of Schools of Public Health in the European Region
DOOHICHE: Democratically-Open Outstanding Hybrid of Internet-aided Computer-aided, and Human-aided Education
MPH: Master of Public Health.
